# Identification and Characterization of the Cognate Anti-Sigma Factor and Specific Promoter Elements of a *T. tengcongensis* ECF Sigma Factor

**DOI:** 10.1371/journal.pone.0040885

**Published:** 2012-07-16

**Authors:** Jingfang Liu, Jie Li, Zhenfang Wu, Huadong Pei, Jian Zhou, Hua Xiang

**Affiliations:** State Key Laboratory of Microbial Resources, Institute of Microbiology, Chinese Academy of Sciences, Beijing, People’s Republic of China; Arizona State University, United States of America

## Abstract

Extracytoplasmic function (ECF) σ factors, the largest group of alternative σ factors, play important roles in response to environmental stresses. Tt-RpoE1 is annotated as an ECF σ factor in *Thermoanaerobacter tengcongensis*. In this study, we revealed that the *Tt-tolB* gene located downstream of the *Tt-rpoE1* gene encoded the cognate anti-σ factor, which could inhibit the transcription activity of Tt-RpoE1 by direct interaction with Tt-RpoE1 via its N-terminal domain. By *in vitro* transcription assay, the auto-regulation ability of Tt-RpoE1 was determined, and band shift assay showed that Tt-RpoE1 preferred to bind a fork-junction promoter DNA. With truncation or base-specific scanning mutations, the contribution of the nucleotides in −35 and −10 regions to interaction between Tt-RpoE1 and promoter DNA was explored. The promoter recognition pattern of Tt-RpoE1 was determined as 5′ tGTTACN_16_CGTC 3′, which was further confirmed by *in vitro* transcription assays. This result showed that the Tt-RpoE1-recognized promoter possessed a distinct −10 motif (−13CGTC−10) as the recognition determinant, which is distinguished from the −10 element recognized by σ^70^. Site-directed mutagenesis in Region 2.4 of Tt-RpoE1 indicated that the “D” residue of DXXR motif was responsible for recognizing the −12G nucleotide. Our results suggested that distinct −10 motif may be an efficient and general strategy used by ECF σ factors in adaptive response regulation of the related genes.

## Introduction

As an essential component of RNA polymerase (RNAP), bacterial sigma (σ) factors play an important role during the initiation of transcription by specifically recognizing and binding to promoter DNA elements [1], [2], [3]. The σ factors can be grouped into two families: the σ^70^ and σ^54^ families [1], [4]. Most of the σ factors belong to the σ^70^ family, which can be structurally and functionally subdivided into four groups [1], [2], [5]. Group I σs are essential housekeeping σs, e.g. *Escherichia coli* (*E. coli*) σ^70^ and *Thermus aquaticus* (*Taq*) σ^A^, which contain four domains designated σ_1_ to σ_4_ [5]. The other groups (II to IV) are alternative σ factors, which can substitute for the primary σ factors to redirect RNAP to initiate the transcription of some specific genes that respond to cell differentiation or environmental stresses [1]. Extracytoplasmic function (ECF) σ factors comprise group IV, the largest and most diverse subfamily, which contains only two domains, σ_2_ and σ_4_. This family regulates the transcription of genes involved in cell envelope functions, including periplasmic stress, heat shock response, iron transport, metal ion efflux, and alginate secretion [1].

A common feature of most ECF σ factors is their regulation by a co-transcribed trans-membrane anti-σ factor. The best understood archetypes include *E. coli* σ^E^ and *Bacillus subtilis* σ^W^ [6], [7], [8]. Direct interaction between ECF σ factor and the intracellular domain of anti-σ factor will prevent the ECF σ factor from binding to RNAP and promoters under normal conditions [9]. Another feature of ECF σ factors is their ability to auto-regulate their own expression and to induce expression of a group of genes synchronously in response to a particular stress [1].

Genome sequencing has revealed numerous ECF σ factors existing in a wide variety of bacteria including many pathogens, and in many organisms, the different ECF σ factors often outnumber all other σ factors combined. For example, *Streptomyces coelicolor*, living in a hostile and changing soil environment, has 55 ECF σ factors among a total of 65 σ factors [1]. It is believed that there is a rough correlation between the apparent complexity of the environment and the number of alternative σ factors [10]. Thus, identification of target genes (regulon) dependent on ECF σ factors is an important way to learn their physiological functions under stressful environmental conditions. Because identification of auto-regulated promoters of ECF σ factors provides clues for predicting the corresponding regulon in the genome [1], it becomes an important strategy to explore the mechanism of environmental adaptation in organisms. Importantly, most alternative σ factors are more selective and recognize more highly conserved promoter motifs than the housekeeping σ factors [11], which makes it more amenable to predict their promoters. Rhodius and his colleagues have predicted the promoter of *E. coli* σ^E^ by different bioinformatic analyses [10], [12]. Helmann and his coworkers developed the “promoter consensus search” method and succeeded in predicting the regulons of σ^W^, σ^X^ and σ^M^ in *B. subtilis* [13], [14], [15], [16]. However, the functional relevance of the bases in the −35 and −10 regions remains in question, and the amino acid residues in the ECF σ factors that mediate recognition of the two regions are still not clear.

*Thermoanaerobacter tengcongensis* belonging to the phylum Firmicutes, is an anaerobic, rod shaped, and low G+C content (33%) thermophilic bacterium, which was isolated from a freshwater hot spring in China and grows between 50–80°C, with an optimum temperature of approximately 75°C [17]. Complete genome sequencing of *T. tengcongensis* revealed seven ECF σ factors (TTE0323, TTE0872, TTE1557, TTE1559, TTE2178, TTE2311, and TTE2400, named Tt-RpoE1 to Tt-RpoE7, respectively), which were predicted to contribute to the adaptation of this thermophile to a high temperature environment [18]. In this work, we identified the cognate anti-σ factor of Tt-RpoE1, and determined the specific promoter sequence recognized by Tt-RpoE1. We clarified the functionally relevant bases in the −35 and −10 regions of the promoter using binding affinity analysis between Tt-RpoE1 and the promoter with scanning mutations, which was also confirmed by *in vitro* run-off transcription analysis. In addition, we identified that the “DXXR” motif in Region 2.4 of Tt-RpoE1 is responsible for recognizing the −12G nucleotide of −10 element. Our studies indicate that a specific element (−13 CGTC −10) in the −10 region of the promoter recognized by Tt-RpoE1 distinguishes it from the σ^70^ promoter, and that may be a general strategy used by ECF σ factor to regulate its extracytoplasmic functions.

## Results

### Analysis of the *Tt-rpoE1* Gene Cluster and the Possible Anti-σ Factor of Tt-RpoE1

**Figure 1:**
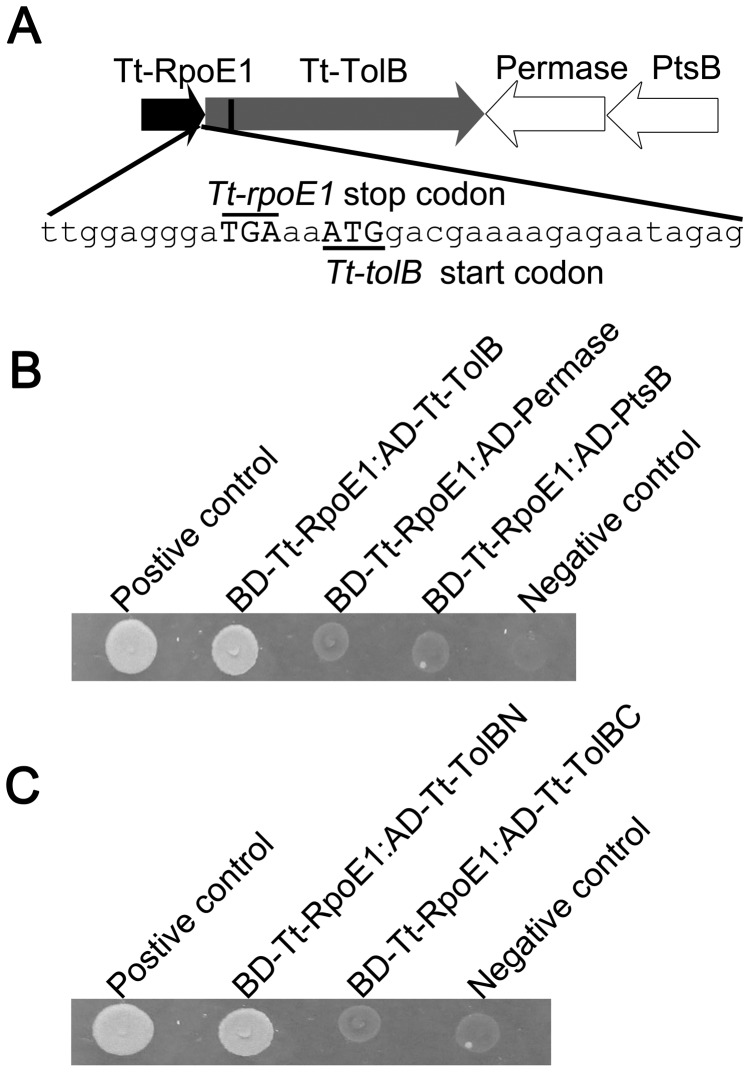
The interaction between *T. tengcongensis* ECF σ factor Tt-RpoE1 and its putative anti-σ factor Tt-TolB. (**A**). Organization of the genes encoding Tt-RpoE1, Tt-TolB and two other proteins (Permease and PtsB). The vertical black line in Tt-TolB indicates the predicted membrane-spanning domain. Partial sequence including the stop codon of *Tt-rpoE1* and the start codon of *Tt-tolB* is shown. (**B**). Y2H analysis of the interaction between Tt-RpoE1 and Tt-TolB and between Tt-RpoE1 and the other two proteins. (**C**). The interaction between Tt-RpoE1 and the N-terminal or the C-terminal region of Tt-TolB.

Genomic sequence analysis showed that the *Tt-tolB* gene is located immediately downstream of the *Tt-rpoE1* gene, and there are two other genes located downstream but with opposite direction (Fig. 1A). There were only 2 base pairs between the stop codon of *Tt-rpoE1* and the start codon of *Tt-tolB* (Fig. 1A), consistent with co-transcription of the regulon gene. The product of *Tt-tolB* contained a 23-amino acid (residues 39–61) trans-membrane domain predicted by the program SOSUI (http://bp.nuap.nagoya-u.ac.jp/sosui/sosui_submit.html). Based on the features of Tt-TolB, we hypothesized that it was the potential anti-σ factor of Tt-RpoE1. As such kind of anti-σ factor usually harbours a trans-membrane domain and could tightly bind to the ECF σ factor [19], we first employed a yeast two-hybrid (Y2H) analysis to assay for direct interaction between Tt-RpoE1 and Tt-TolB, as well as those between Tt-RpoE1 and the products of the other two downstream genes. We cloned *Tt-rpoE1* into pGBKT7 to obtain a DNA-binding domain (BD) fusion construct and then cloned all the other genes of this cluster into pGADT7 to obtain activation domain (AD) fusions. As shown in Fig. 1B, the co-transformant containing BD-Tt-RpoE1/AD-Tt-TolB could grow on the selective media and activate the *lacZ* reporter gene, indicating a specific interaction between Tt-RpoE1 and Tt-TolB, whereas no interaction was observed between Tt-RpoE1 and the other two gene products. In many cases of RpoE-like ECF σ factors, it was sequestered by the intracellular domain of its cognate anti-σ factors [1], [7], [20]. To examine whether this is the case for Tt-TolB, we subcloned the coding sequences of the N-terminal (residues1–39) and C-terminal (residues 62–645) domains of *Tt-tolB* into pGADT7 and found that the N-terminal domain of Tt-TolB interacted with Tt-RpoE1 specifically (Fig. 1C). These results further suggested that Tt-TolB might be the anti-σ factor of Tt-RpoE1.

### Tt-RpoE1 Activates its Own Gene Transcription *in vitro*

**Figure 2:**
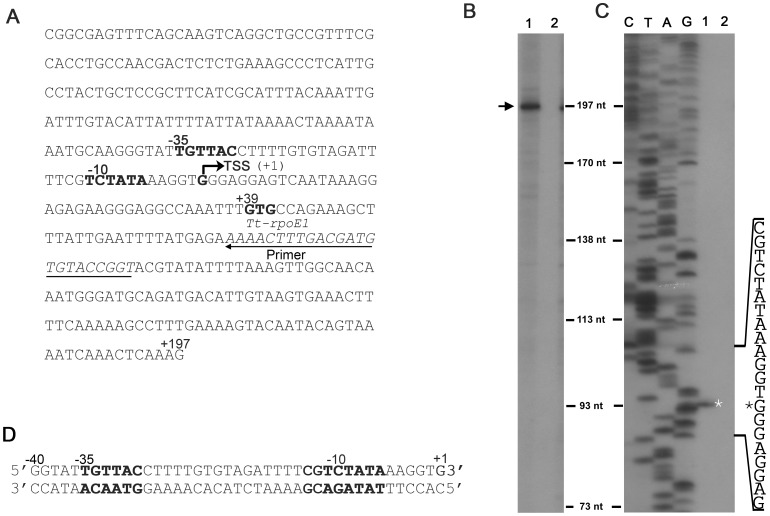
Analysis of Tt-RpoE1 recognition of its own promoter sequence by *in vitro* transcription and primer extension assays. (**A**). Sequence of the *in vitro* run-off transcription which includes 218 bp upstream and 159 bp of *Tt-rpoE1* gene. (**B**). *In vitro* run-off transcription of *Tt-rpoE1* template (**A**) by *E. coli* core RNAP with (lane 1) or without (lane 2) Tt-RpoE1. The transcription product is indicated with an arrow. (**C**). Mapping of the transcriptional start site (TSS) of *Tt-rpoE1* by primer extension, in which RNA was isolated from *in vitro* transcription reactions with (lane 1) or without (lane 2) Tt-RpoE1. The TSS is marked by an asterisk. Lanes C, T, A, and G are the DNA sequencing ladder corresponding to the primer extension results. The relevant sequence is shown at the side. The *Tt-rpoE1* promoter region (the deduced −10 and −35 regions), the TSS and the putative translation start codon (+39) are all indicated in (**A**). The position of the primer used for primer extension and DNA sequencing is underlined. (**D**). Sequence of the double-stranded parental probe for the following EMSA assay.

Many ECF σ factors are able to recognize their own promoter and thereby auto-regulate their own gene expression [14], [21]. To test if Tt-RpoE1 can activate its own gene transcription, we used a PCR product containing 159-base pair (bp) (+39 ∼ +197) of *Tt-rpoE1* gene and a 218 bp upstream region as the template and analyzed the ability of Tt-RpoE1 to recognize it by *in vitro* run-off transcription reconstitution assays (Fig. 2A). Indeed, with the *E. coli* core RNAP, Tt-RpoE1 could activate the *Tt-rpoE1* promoter *in vitro*, resulting in a significant transcript (∼197nt) (lane 1, Fig. 2B), indicating the transcription start site is located about 39 bp upstream of the GTG start codon of *Tt-rpoE1*. In the control experiment when Tt-RpoE1 was omitted, there was no transcription product generated (lane 2, Fig. 2B), indicating the *T. tengcongenesis* sigma factor is required to initiate transcription from its own promoter.

To identify the recognition sequence in the *Tt-rpoE1* promoter, the Tt-RpoE1-dependent start site was further confirmed by primer extension analysis with the *in vitro* transcripts. As shown in Fig. 2C, the transcription start site (TSS) was located at the “G” exactly 39 bp upstream from the translational start codon. Based on the TSS, we deduced the location of the −35 region (−35 TGTTAC −30) and −10 region (−11 TCTATA −6), which are spaced apart by 18 bp (Fig. 2A). This putative promoter sequence was subjected to comprehensive interaction and mutagenesis analyses for further characterization of the recognition determinant.

### Interaction between Tt-RpoE1 and The *Tt-rpoE1* Promoter in Fork-junction Structure

As the promoter sequence of *Tt-rpoE1* was deduced, we then investigated how Tt-RpoE1 binds to its own promoter sequence as an auto-regulated ECF σ factor. It has been previously demonstrated that free σ^70^ could not bind to promoter DNA due to the inhibition of its subdomain σ_1.1_ [22], [23]. Because ECF σ factors lack this N-terminal subdomain σ_1.1_ [24], we predicted Tt-RpoE1 could bind its promoter sequence without core RNAP. To test this prediction, we conducted binding studies with an electrophoretic mobility shift assay (EMSA). First, the double-stranded probe (T+1/B+1) corresponding to −40 to +1 bp of the promoter region was used (Fig. 2D), but only weak binding between the double-stranded promoter region and Tt-RpoE1 could be observed when the protein concentration was much high (25 µM, data not shown).

**Figure 3:**
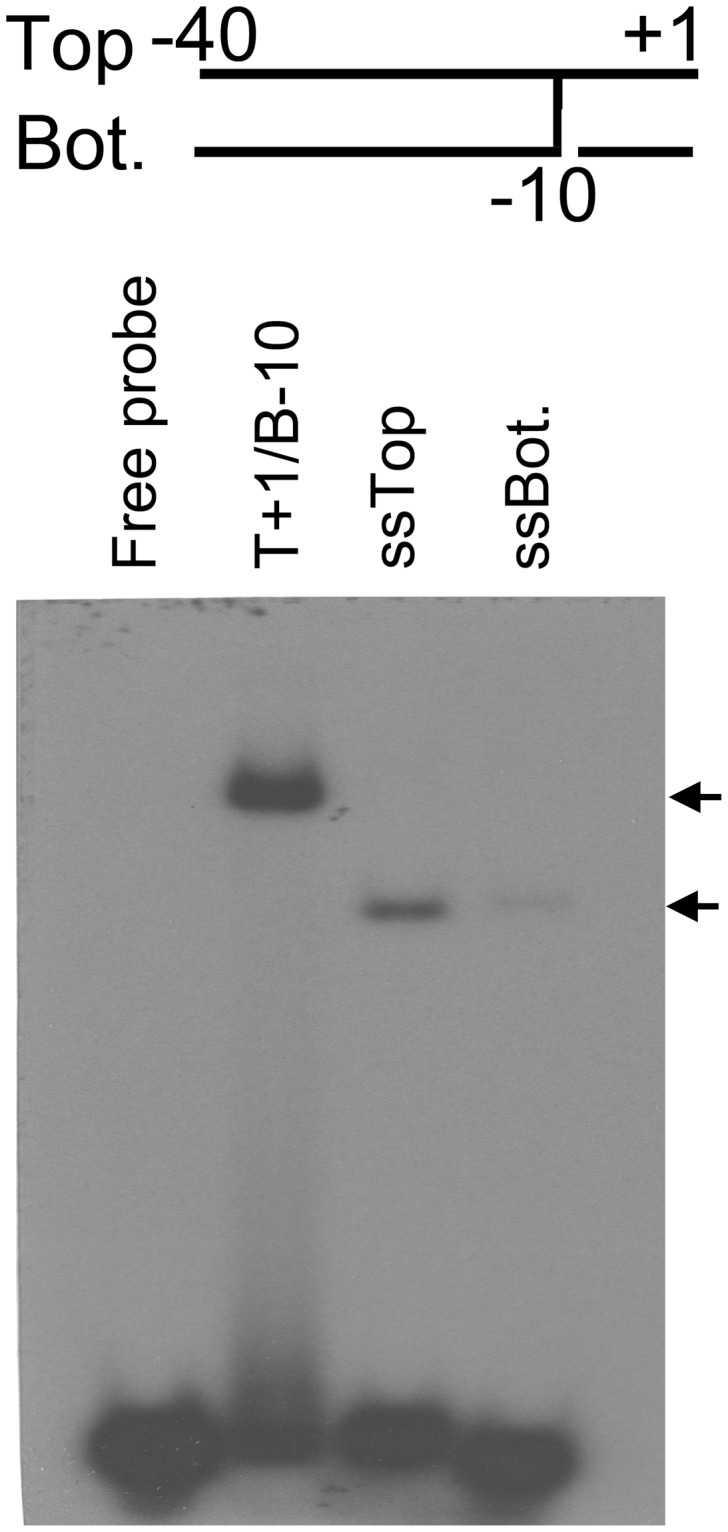
The interaction between Tt-RpoE1 and different promoter DNA structures. The structure of parental probe is provided at the top. EMSA results of 5 µM Tt-RpoE1 protein binding with single-stranded (ssTop, ssBot.) or fork-junction structure promoter DNA(T+1/B−10); the vertical line indicates the terminal base-pair on the strands used in fork-junction probe. Free probe (T+1/B−10) was loaded as a negative control. The arrows indicate complexes formed by Tt-RpoE1 and the different probes.

Previous studies showed that σ^54^ and σ^70^ holoenzymes tightly bind to fork-junction promoter DNA [25], because this structure partially mimics the open state of the promoter DNA, which includes a duplex upstream of −10 region and a single-stranded −10 region [26]. Therefore, we examined the interaction between Tt-RpoE1 and the fork-junction probe of the promoter DNA, which was obtained by “cutting back” the bottom strand from B+1 to B−10 (Fig. 3, lane T+1/B−10). The result was in striking contrast to the weak binding of duplex probe, there was a strong preference for Tt-RpoE1 to bind to the fork-junction probe. This finding is likely because the −10 region on the non-template strand became accessible when the template (bottom) strand was cut back. It was not surprising to observe only a weak interaction between Tt-RpoE1 and the non-template single-stranded DNA (ssTop, Fig. 3), which implied that the double-stranded −35 element is also important for Tt-RpoE1 binding to promoter DNA. Notably, a much weaker band formed by Tt-RpoE1 and the template strand (ssBot., Fig. 3) was also found at the same position. These results clearly indicated that Tt-RpoE1 could efficiently bind to the *Tt-rpoE1* promoter, consistent with the result of the run-off transcription assay, and showed that *Tt-rpoE1* gene would be auto-regulated in *T. tengcongensis*.

We also detected the interaction between the promoter and the reconstituted holoenzyme (formed by Tt-RpoE1 and *E.coli* core RNAP). It exhibited little difference in binding efficiency to that of Tt-RpoE1 alone (data not shown), implying that high concentration of Tt-RpoE1 (5 µM) could decrease the “activation effect” of core RNAP on the binding affinity [27]. Thus, we omitted the core RNAP in the following EMSA reaction. On the other hand, since *T. tengcongensis* grows at an optimum temperature of approximately 75°C, we also assayed the interaction between Tt-RpoE1 and the fork-junction promoter DNA at different temperatures from 25 to 80°C. Tt-RpoE1 exhibited a similar binding affinity to promoter DNA from 25 to 55°C, indicating that it functioned very well at a wide range of temperatures (data not shown). However, when performed at 60 to 80°C, the fork-junction promoter DNA partially melted (data not shown), which is not favorable for studying the interaction between Tt-RpoE1 and the promoter DNA. Therefore, in order to mimic the Tt-RpoE1/promoter interaction in an open complex of transcription initiation *in vivo*, we performed the EMSA experiment at 25°C to investigate the interaction between Tt-RpoE1 and the fork-junction promoter DNA in the following experiments.

### Effect of Tt-TolB as an Anti-sigma Factor on the Activity of Tt-RpoE1

**Figure 4:**
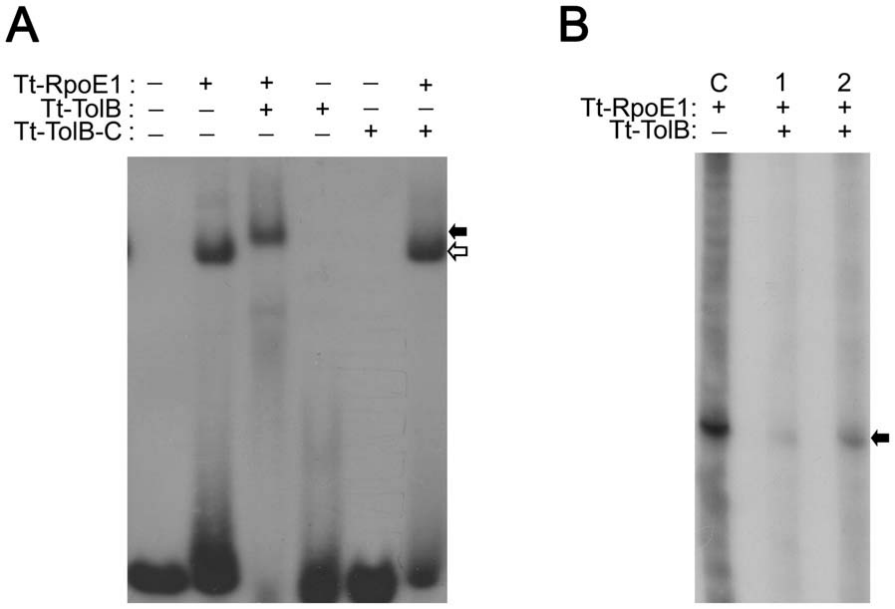
The effect of putative anti-σ factor Tt-TolB on the activity of Tt-RpoE1. (**A**). The effect of Tt-TolB on the interaction between Tt-RpoE1 and fork-junction structure promoter DNA (T+1/B−10). The indicated proteins were added (+) in an EMSA reaction at a concentration of 5 µM. The solid arrow indicates the supershifted complex formed by Tt-RpoE1, Tt-TolB, and the promoter, and the open arrow indicates the complex formed by Tt-RpoE1 and the promoter. (**B**). The effect of Tt-TolB on *in vitro* transcription of Tt-RpoE1. Lane 1. Tt-TolB was added into the transcription system at the same time with Tt-RpoE1. Lane 2. Tt-TolB was added into the transcription system after Tt-RpoE1, *E.coli* core RNAP and promoter DNA being incubated (see materials and methods). Lane C, the *in vitro* transcription system without Tt-TolB. The solid arrow indicates the products of transcription.

To investigate if the proposed anti-σ factor Tt-TolB would affect the interaction between Tt-RpoE1 and its promoter, we added equimolar concentrations of Tt-TolB or its C-terminal Tt-TolB-C (N-terminal of Tt-TolB is not used as it is too short to be purified), respectively, to the EMSA reaction with Tt-RpoE1, using the fork-junction promoter DNA (T+1/B−10) as a template. As shown in Fig. 4A, when Tt-TolB was added into the EMSA reaction system, a supershifted complex larger than the Tt-RpoE1/promoter complex was formed. However, adding Tt-TolB-C into the EMSA reaction system did not lead to the formation of a larger complex, and neither Tt-TolB nor Tt-TolB-C alone could bind to the fork-junction promoter DNA. The results suggested that the larger complex was formed by a direct interaction between Tt-RpoE1 and Tt-TolB (Fig. 4A), and the interaction was mediated by the N-terminal domain of Tt-TolB, consistent with Y2H result in Fig. 1C. We also tested if Tt-TolB affected the transcription of Tt-RpoE1 *in vitro*. Equimolar concentration of Tt-TolB to Tt-RpoE1 was added into the transcription system. In lane 1 of Fig. 4B, Tt-TolB was added into reaction at the same time with Tt-RpoE1, and in lane 2, Tt-TolB was added with NTP together, after Tt-RpoE1, promoter DNA and RNAP were incubated together for short time (see materials and methods). The transcription products decreased in both of them, which indicated that Tt-TolB could inhibit the transcription of Tt-RpoE1 by interaction with it. The product in lane 1 was less than that in lane 2, which might be due to Tt-TolB competing for Tt-RpoE1 with RNAP. When Tt-TolB was added at the same time with RNAP and Tt-RpoE1, it decreased the RNAP binding to Tt-RpoE1 more than Tt-TolB added into system later. Together with Y2H results, these results confirmed that Tt-TolB was the anti-σ factor of Tt-RpoE1 and that it interacted with the ECF σ factor Tt-RpoE1 via direct interaction.

### Determination of the **−**35 and **−**10 Regions in the Tt-RpoE1-recognized Promoter

**Figure 5:**
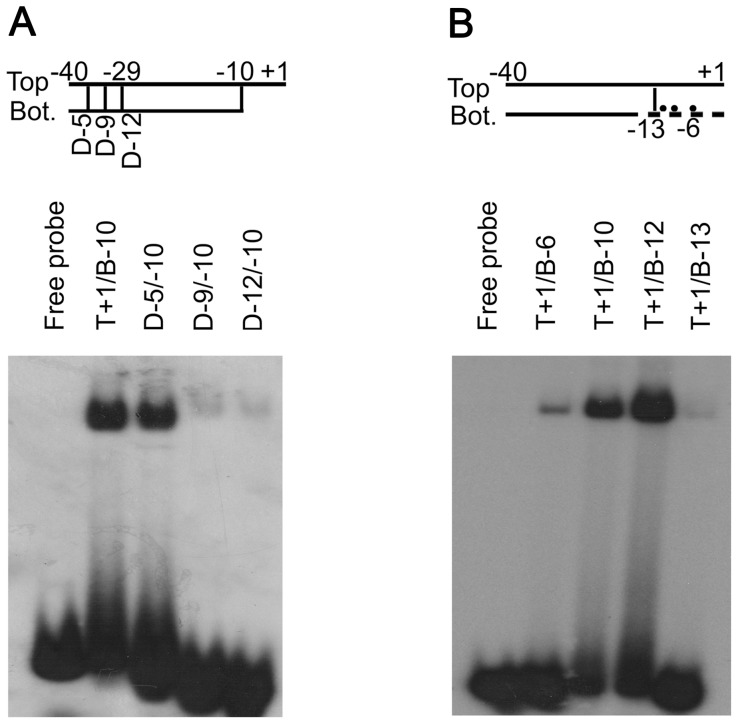
The effects of truncation in the −35 and −10 regions on the interaction between Tt-RpoE1 and promoter DNA. (**A**). EMSA results of truncation in the −35 region. The structure of parental probe is provided at the top, and vertical lines indicate truncated positions in the double-stranded region. Both the top and bottom strands were truncated from −40 to different positions indicated at the left of the fork-junction probe. The 3′ terminus of top strand was kept at +1, and the 5′ terminus of the bottom strand was kept at −10. (**B**). EMSA results of truncations in the −10 region. The structure of parental probe is provided at the top. The T+1 was the top strand for all the probes, and the bottom strand was truncated from B+1 to different positions as indicated on the right of the bottom strand. The dots denote the terminal bases in the bottom strands in fork-junction probes. The protein concentration was 5 µM in all of the following experiments.

To experimentally determine the sequences of the promoter recognized by Tt-RpoE1, we analyzed the interaction between Tt-RpoE1 and different fork-junction promoter probes with truncations in the putative −10 or −35 regions. For the −35 region using T+1/B−10 as the parental probe, we cut back the double strands from −40 to different positions (marked by vertical lines, Fig. 5A; for the sequences, see Table S1). Notably, the 5 bp truncation from −40 to −36 (D-5) had little effect on the binding strength between the promoter and Tt-RpoE1, but further truncation from −35 to −32 (D-9) resulted in a strong decrease in binding affinity. Tt-RpoE1 binding was almost abolished when the −35 region was removed (D-12) (Fig. 5A). This result suggested that the removal of 4 bp from −35 to −32 of the −35 region eliminated determinants of recognition. Therefore, the 4-bp sequence was very important for promoter recognition of Tt-RpoE1, and likely the −10 region could not be recognized and bound by Tt-RpoE1 without the −35 region.

For the −10 region using T+1/B+1 as the parental probe, the top strand (T+1) was left intact and the bottom strand was cut back from +1 to different positions (Fig. 5B). As shown in Fig. 5B, the binding strength became stronger with more non-template sequences of the −10 element exposed. The binding was the strongest with the nucleotide −11T in the non-template strand exposed (lane T+1/B−12), but at position B−13, the binding affinity decreased significantly (lane T+1/B−13, Fig. 5B). These results indicated that the −12 position remaining base-paired was required for Tt-RpoE1 binding, which might be the similar situation of the interaction between σ^54^ and its promoter [25]. These data also confirmed our initial prediction of the −35 and −10 regions.

### Identification of the Specific Recognition Determinants in the Tt-RpoE1-recognized Promoter

Although the truncation results of the −35 and −10 regions gave clues to the location of promoter recognition by Tt-RpoE1, the contribution of each nucleotide to recognition was still unresolved. To precisely define the conserved nucleotides needed for recognition by Tt-RpoE1, we performed scanning mutagenesis by nucleotide substitutions between G-C and A-T in the −35 and −10 regions.

**Figure 6:**
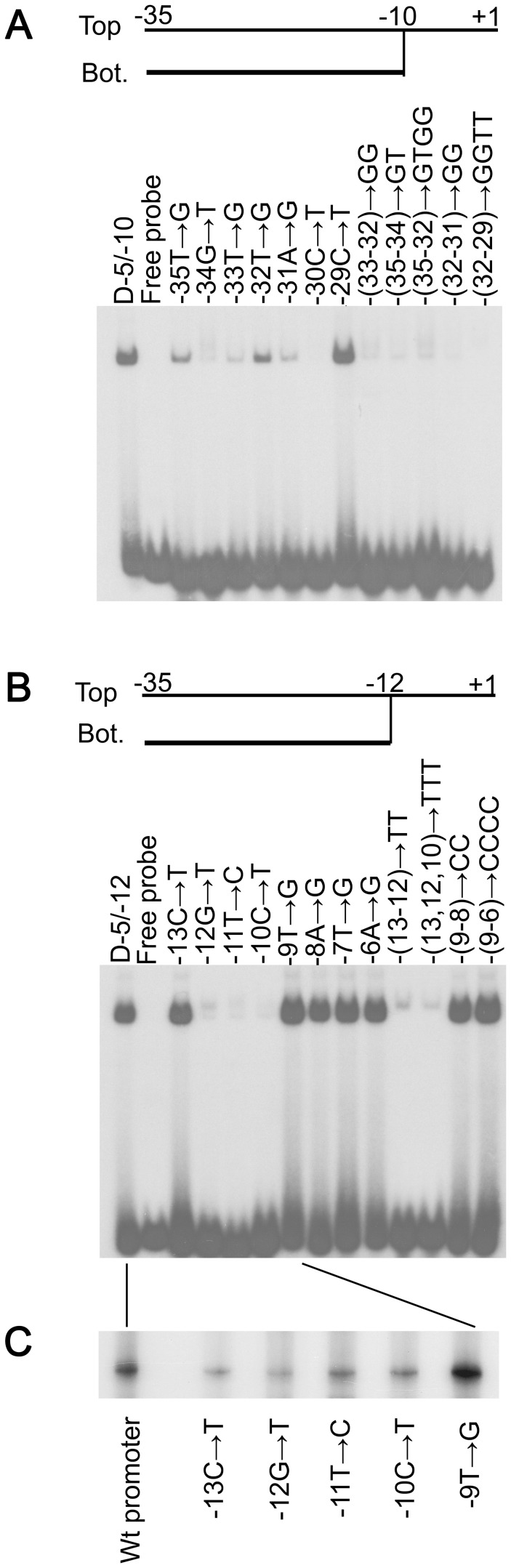
The effect of scanning substitutions in the −35 and −10 regions on the activity of Tt-RpoE1. (**A & B**). EMSA results of substitutions in the −35 (**A**) and −10 (**B**) regions. The D−5/−10 or D−5/−12 fork-junction structure of promoter DNA was used as the parental probe respectively. The substitutions were made both on the top and opposite positions of the bottom strands in the duplex part as indicated. (**C**). The effect of a subset of substitutions in the −10 region on *in vitro* transcription of Tt-RpoE1.

For the −35 region using D−5/−10 as the parental probe, the nucleotides were substituted in top and bottom strands simultaneously. The EMSA results were shown in Fig. 6A. Among the seven nucleotide substitutions (from −35 to −29), substitution of nucleotide −30C to T abolished Tt-RpoE1 binding, and substitutions at positions −34, −33 and −31 also significantly decreased the binding, whereas substitution of −29C to T had little effect on the binding affinity. Not surprisingly, double, triple and quadruple substitutions severely affected the binding affinity, likely due to cumulative effects. The result of four substitutions (from −35 to −32) was consistent with the truncation result of D-9. Interestingly, the double substitutions −32TA−31 to GG led to a more significant decrease than the other two double mutations or even the 4-bp substitutions from −35 to −32, which suggest that −32TA−31 dinucleotide together play a key role in the interaction between Tt-RpoE1 and promoter DNA. Both the results of substitutions at −30C and −32TA−31 were consistent with abolition of binding in the 4-bp substitutions from −32 to −29. Thus, we concluded that the −35 element recognized by Tt-RpoE1 contained the following sequence: tGTTAC (with important nucleotides capitalized).

For the −10 region using the strongest binding structure (D−5/−12) as the parental probe, scanning mutations were made from −11 to −6 on the top strand, while the −13 (C/G) and −12(G/C) base pairs were substituted in both top and bottom strands. The EMSA results were shown in Fig. 6B. Single substitutions at −12G to T, −11T to C, and −10C to T almost abolished Tt-RpoE1 binding, whereas substitutions at position −13 and each nucleotide in −9TATA−6 had little effect on the binding affinity. In addition, the double substitution of −13CG−12 to TT and triple substitution of −13C−12G−10C to TTT also significantly decreased the binding, which further suggested that −12 GTC −10 was the determinant of Tt-RpoE1 recognition. In contrast, double substitution of −9TA−8 to CC and quadruple substitution of −9TATA−6 to CCCC had little effect on binding affinity, indicating that the TATA region (from −9 to −6) downstream of GTC may not contribute to the recognition. Based on these scanning mutagenesis results, we propose that the core sequence recognized by Tt-RpoE1 at the −10 region was determined to be −12GTC−10.

We also carried out *in vitro* transcription assays to test if those important nucleotides determined by EMSA would affect the transcription activity of Tt-RpoE1. Since the structure of the complex of *E. coli* σ^E^_4_ and its −35 element has been determined [28], which provided some clues for our results in −35 element determinant, here, we only took a subset of the mutations at the −10 region of the promoter DNA as templates to detect their effect on transcription activity of Tt-RpoE1. For those promoters substituted from −13 to −9, it was clear that the substitutions at −12GTC−10 decreased transcription significantly (Fig. 6C), which confirmed the EMSA results that the −12GTC−10 was indeed the recognition determinants at the −10 region. For the substitution at −9T, it led to a slight increase of transcription, which was consistent to the EMSA results (Fig. 6B). Interestingly, substitution at −13C also decreased the transcription, which was different from the EMSA results, where binding was similar to the wild type (wt) promoter. The observed data suggested substitution at −13C may affect the interaction between RNAP and promoter DNA.

Based on the results of EMSA and transcription analysis, the determinant sequence (−35 and −10 regions) for the promoter recognized by Tt-RpoE1 could be identified as 5′ tGTTACN_16_CGTC 3′.

### Identification of Residues in Tt-RpoE1 Potentially Involved in Recognition of the **−**10 Region

**Figure 7:**
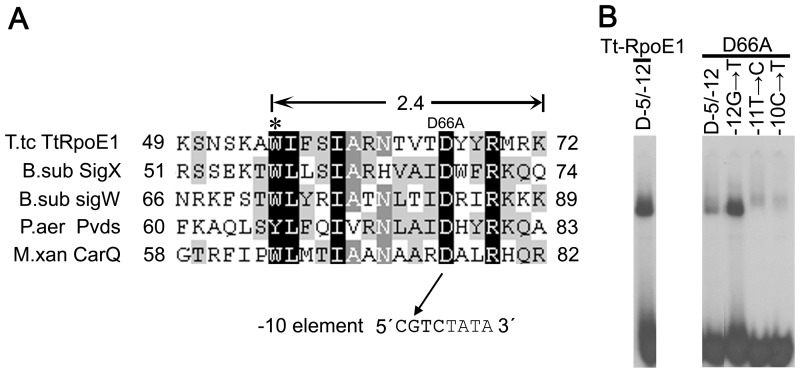
The effects of single amino acid substitution in Region 2.4 of Tt-RpoE1 on the interaction between Tt-RpoE1 and promoter DNA. (**A**). Alignment of the amino acid sequence of Regions 2.4 in group IV ECF σ factors. The numbers at each end of the sequence indicate the amino acid position. The substitutions in Tt-RpoE1 used in this study are shown. The asterisk indicates the conserved amino acids of the melting residues of σ^70^. Species abbreviations and GenBank accession numbers of their proteins are as follows: *Thermoanaerobacter tengcongensis* (T.tc), Tt-RpoE1 (NP_622011.1); *Bacillus subtilis* (B.sub), SigX (NP_390191.2), SigW (NP_388054.1); *Pseudomonas aeruginosa* (P.aer), PvdS (NP_251116.1); *Myxococcus Xanthus* (M.xan), CarQ (YP_632266.1). The recognition between D66 and −12G is indicated by arrow. (**B**). The effects of single amino acid substitution in Region 2.4 of Tt-RpoE1 on the interaction between Tt-RpoE1 and wild-type (D−5/−12) or mutated promoter DNA. Tt-RpoE1 D66A uniquely suppressed single nucleotide changes at position −12G.

The studies above showed that Tt-RpoE1 recognized a specific −10 element (−13CGTC−10) which is distinct from that recognized by σ^70^ (TATAAT) [29]. To assess the importance of particular amino acid residues for Tt-RpoE1-specific promoter recognition in the −10 region, we employed alanine substitution mutagenesis to the Region 2.4 of Tt-RpoE1. For σ^70^ family, Region 2 has been implicated in recognition of −10 regions [4], [30]. Selection of amino acid residues for substitution was based on sequence alignments among group IV ECF σ factors (Fig. 7A). We substituted three residues (D66, Y67, R69) in the conserved motif “DXXR” based on the sequence alignment shown in Fig. 7A. Of the three alanine substitution mutations, only D66A strongly decreased the binding affinity to the wt promoter (Fig. 7B); while the binding affinity of Y67 and R69A remained (data not shown). We then tested whether this alanine mutation could suppress the promoter defects caused by base changes at −12GTC−10 in the promoter, as it has been shown that −13C to T had no effect on the Tt-RpoE1-promoter interaction (Fig. 6B). Interestingly, D66A could cure the defect caused by change at −12G position, but not at the −11T and −10C (Fig. 7B). Thus, D66 might contribute to the recognition of the −12G of the promoter.

**Table 1:** Plasmids used in this study.

Plasmids	Description	Sources or references
pET−28a	Kan^r^, expression vector with His-tag coding sequence	Novagen
pET−23b	Amp^r^, expression vector with His-tag coding sequence	Novagen
P28Tt-RpoE1	pET−28a derivative for expression of the Tt-RpoE1	this work
P28Tt-TolB	pET−28a derivative for expression of the Tt-TolB	this work
P28Tt-TolB-C	pET−28a derivative for expression of the C-terminal domain of Tt-TolB	this work
pGBKT7(BD)	Yeast two-hybrid DNA-binding domain vector	clontech
pGADT7(AD)	Yeast two-hybrid activation domain vector	clontech
BD-Tt-RpoE1	pGBKT7 derivative for expression of the Tt-RpoE1	this work
AD-Tt-TolB	pGADT7 derivative for expression of the Tt-TolB	this work
AD-Tt-TolBN	pGADT7 derivative for expression of the N-terminal domain of Tt-TolB	this work
AD-Tt-TolBC	pGADT7 derivative for expression of the C-terminal domain of Tt-TolB	this work
AD- Permase	pGADT7 derivative for expression of the Permase	this work
AD- PtsB	pGADT7 derivative for expression of the PtsB	this work

## Discussion

In this work, we addressed the function of Tt-RpoE1, one of seven ECF σ factors annotated in the genome of *T. tengcongensis*. Y2H and EMSA results showed that Tt-TolB, the cognate downstream gene product of Tt-RpoE1, interacted with Tt-RpoE1 via its N-terminal domain. Tt-TolB also inhibited the transcription of Tt-RpoE1. These results demonstrated that Tt-TolB (TTE0322) was the anti-sigma factor of Tt-RpoE1. While TTE0322 was originally annotated as Tt-TolB for containing a conserved domain of TolB, a periplasmic component of the Tol biopolymer transport system [31], we now update the function of Tt-TolB to be an anti-sigma factor of Tt-RpoE1. Combined with these findings and that Tt-RpoE1 recognized its own promoter and initiated transcription, we confirmed Tt-RpoE1 functionally as an ECF σ factor.

**Table 2:** Partial oligonucleotides used in this study.

Names	Sequences(5′ to 3′)*	Purposes
P1	GCGAATTCAGCTTTATTGAATTTTATGAG	P1/P2:BD-Tt-RpoE1
P2	TCGGATCCTCATCCCTCCAAACATTT	
P3	TCCTCGAGTCATCCCTCCAAACATTT	P1/P3: p28Tt-RpoE1
P4	ATGAATTCCGGCGAGTTTCAGCAAGT	P4/P2: pPTt-RopE1-T
P5	AGGAATTCGACGAAAAGAGAATAGAG	P5/P6: AD-Tt-TolB
P6	TTGGATCCGTATTAAAACCTGCCCTT	
P7	CTCTCGAGTATCTTTTTCCATCTGTT	P5/P7: AD-Tt-TolBN
P8	AGGAATTCCAAGATAATTTAATAACA	P8/P6: AD-Tt-TolBC
P9	ACGAATTCATGGACGAAAAGAGAATAGA	P9/P10: p28Tt-TolB
P10	CTCTCGAGGTATTAAAACCTGCCCTT	P8/P10: p28Tt-TolB-C
P11	TGGAATTCAGTACTAGCTCTTTGATTTT	P11/P12: AD-Permase
P12	TGGGATCCGGCCTGCCTTAGTTGATG	
P13	CGGAATTCGTGGAAATAGAGCTTAAAAA	P13/P14: AD-PtsB
P14	CGGGATCCTCACTTTATCATCTCCTTTA	P4/P15 for transcription template
P15	CTTTGAGTTTGATTTTAC	
P16	ACCGGTACACATCGTCAAAGTTTT	For primer extension
P17	CCGAAATACTGTGACAGCCTATTACAGAATGAGGA	P17/18: D66A of p28Tt-RpoE1
P18	TCCTCATTCTGTAATAGGCTGTCACAGTATTTCGG	
P39	TTTTGTGTAGATTTTTGTCTATAAAGGTGGGAGGAG	P39/40:mutation at −13C of transcription template
P40	CTCCTCCCACCTTTATAGACAAAAATCTACACAAAA	
P41	TTTGTGTAGATTTTCTTCTATAAAGGTGGGAGGAGT	P41/42:mutation at −12G of transcription template
P42	ACTCCTCCCACCTTTATAGAAGAAAATCTACACAAA	
P43	TTGTGTAGATTTTCGCCTATAAAGGTGGGAGGAGTC	P43/44:mutation at −11T of transcription template
P44	GACTCCTCCCACCTTTATAGGCGAAAATCTACACAA	
P45	TGTGTAGATTTTCGTTTATAAAGGTGGGAGGAGTCA	P45/46:mutation at −10C of transcription template
P46	TGACTCCTCCCACCTTTATAAACGAAAATCTACACA	
P47	GTGTAGATTTTCGTCGATAAAGGTGGGAGGAGTCAA	P47/48:mutation at −9T of transcription template
P48	TTGACTCCTCCCACCTTTATCGACGAAAATCTACAC	

* Restriction sites are underlined.

Being an auto-regulated ECF σ factor, the ECF σ factor Tt-RpoE1 was first subjected to investigate the interaction with its promoter. Different from σ^70^, ECF σ factor could bind to double-strand promoter DNA, but it preferred to bind fork-junction structure promoter (Fig. 3). With such structure, we identified the specific promoter sequence recognized by Tt-RpoE1 with scanning mutations, which was further confirmed by *in vitro* transcription assays. The determinant sequence in the Tt-RpoE1-recognized promoter was identified as 5′tGTTACN_16_CGTC3′, which was similar some of the predictions by Staron and coworkers for promoters recognized by RpoE-like (ECF02) σ factors [19].

For the −35 region of the Tt-RpoE1 promoter, we found that −34G, −33T, −30C and −32TA−31 were functionally important for recognition by the ECF σ factor Tt-RpoE1 (Fig. 6A). Substitutions at those positions significantly decreased the binding affinity of Tt-RpoE1. This finding was supported by the structural analysis of the complex of *E. coli* σ^E^_4_ and its −35 element. In that complex, specific protein-DNA base interactions occurred only at three positions of its 7 bp −35 element GGAACTT (underlined): −35G, −34G, and −31C, which were specifically recognized by residues R176, S172 and R171 of *E. coli* σ^E^, respectively [28]. We proposed that −34G and −30 C of −35 element (tGTTAC) of Tt-RpoE1 promoter played the same roles as −35G, −34G, and −31C in the *E. coli* σ^E^ promoter, serving as the key nucleotides to form strong hydrogen bonds or van der Waals interactions with Tt-RpoE1. For the −33T, −32TA−31, they may be similar to the “AA” motif in the −35 element in *E.coli* σ^E^ promoter [28], which plays an essential structure role in the σ^E^_4_^/^promoter interaction. Thus substitution at any one of those nucleotides would disrupt the structure, and affected the Tt-RpoE1/promoter interaction.

For the −10 region, scanning mutagenesis of Tt-RpoE1-recognized promoter indicated that the four nucleotides CGTC (from −13 to −10) are functionally important. Mutations at these bases resulted in loss of Tt-RpoE1 binding affinity and decreasing the transcription activity. However, the “TATA” box downstream of the CGTC motif did not seem to contribute to the interaction between Tt-RpoE1 and its promoter. Taking one of the substitutions −9T to G as example, we have not detected any effect in the EMSA and *in vitro* transcription assay (Fig. 6B, C), indicating that the “TATA” box does not contribute to the recognition of Tt-RpoE1 promoter by Tt-RpoE1. This kind of −10 motif has been found in the −10 regions recognized by several other ECF σ factors, such as PvdS of *P. aeruginosa*, CarQ of *Myxococcus xanthus*, σ^C^ of *Mycobacterium tuberculosis* [32], σ^x^,σ^w^ and σ^M^ in *B. subtilis* [1], [15]. Thus, we proposed that the “CGTC” in the −10 region is a common feature of many promoters recognized by ECF σ factors, especially for those RpoE-like (ECF02) σ factors [19].

We have also identified residues in Tt-RpoE1 contributing to base-specific interactions in the promoter by site-directed mutagenesis employing the same strategy as Koo and his colleagues in their studies [33]. Specifically, loss of the residue interacting with a particular base may suppress the deleterious effects of promoter mutants only at the interacting position(s). Interestingly, mutations at the D66 residue of the conserved motif “DXXR” had strong effect on the Tt-RpoE1/promoter interaction (Fig. 7B). D66A decreased the binding affinity and rescued the defect caused by substitution at −12G. In another ECF σ factor, PvdS from *P. aeruginosa*, the results also suggested that the “D” residue participated in discriminating −10 region contacts [32]. EMSA results showed that −12 position (−13CGTC−10, underlined) kept in base-pair formation was required for recognition. While it is not clear how σ factor recognizes the sequence-specific duplex −10 element [30], here we identified for the first time that the residue “D” of DXXR motif in ECF σ factor recognizes the duplex −10 element (−12G/C).

Notably, we demonstrated that the GC-rich motif in the −10 region recognized by ECF σ factors is significantly different from the consensus sequence (TATAAT) recognized by group I factor σ^70^. This is consistent with their different functions in bacteria. The group I σ factors contain conserved melting residues (F427, Y430, W433 and W434) [5], which makes σ^70^ tolerate a great deal of promoter sequence diversity when directing the transcription of thousands of housekeeping genes. Whereas only one melting residue corresponding to “W” (Fig. 7A, marked by “*”) exists in ECF σ factors. Most recent studies suggest that weak melting capacity of ECF σ factors is consistent with their function acting as local regulators, which are confined to direct the transcription of a more restricted set of promoters in adverse environments [11], [34]. Thus, there is a balance between melting capacity of a σ factor and its promoter specificity. Here, we suggest that recognition of the specific “CGTC” motif in −10 region of Tt-RpoE1-recognized promoter is an important strategy employed by ECF σ factor to strengthen the stringency of its promoter, which enables ECF σ factors respond to environmental stresses in a focused way by regulating a tightly defined regulon [34]. On the other hand, Koo and his colleagues also found that a GC-rich extended −10 motif played important roles in the recognition of group III σ factors σ^28^ of *E.coli*, they proposed that GC-rich promoters may avoid their transcription by the housekeeping σs [33]. It should be the same case for the Tt-RpoE1-recognized promoter. Similar to ECF sigma factor, this GC-rich motif was also recognized by a “DXXR” motif of σ^28^ [33]. Thus, it might be proposed that a distinct −10 element and a “DXXR” motif are the general strategy used by alternative σ factor-dependent regulons to function in the bacterial world, although more structural details for these interactions remain to be investigated in the future.

## Materials and Methods

### Bacteria, Plasmids and Oligonucleotides

*T. tengcongensis* MB4^T^ was routinely grown in modified MB medium at 75°C without shaking [18]. *E. coli* DH5α was used as a host for the cloning experiments, and *E. coli* BL21DE3 (lysS) (Novagen, UK) for overproduction of the recombinant proteins. Both *E. coli* strains were grown in LB medium containing the appropriate antibiotic, ampicillin (Amp, 100 µg/ml) or kanamycin sulfate (km, 50 µg/ml) if necessary. The plasmids and partial oligonucleotides used in this study were described in Tables 1 and 2, respectively.

### DNA Manipulations

The *Tt-rpoE1* (TTE0323) and *Tt-tolB* (TTE0322) coding regions were amplified by PCR from genomic DNA with primers P1/P3 and P9/P10 (Table 2), respectively. Similarly, the DNA fragment encoding the carboxy-terminal portion (residues 62–645) of Tt-TolB protein was obtained by PCR amplification with primers P8/P10 (Table 2). The PCR products were digested and inserted into the corresponding sites of pET28a (Novagen, UK) to generate the expression plasmids p28Tt-RpoE1, p28Tt-TolB and p28Tt-TolB-C respectively. The plasmid p28Tt-RpoE1 was used as a template for the following mutagenesis. Derivatives of Tt-RpoE1 mutated at different residues were amplified with the primers listed in Table 2. The PCR-amplified sequences were verified by DNA sequencing for all of these constructs.

### Yeast Two-hybrid – Assay

Yeast two-hybrid analysis was carried out using the Matchmaker system 3 (Clontech, Palo Alto, CA, USA) according to the manufacturer’s protocol. Genes encoding Tt-RpoE1 (TTE0323) and the other three proteins (TTE0320-0322) including *Tt-tolB* were amplified by PCR from *T. tengcongensis* genomic DNA (for primer sequences, see Table 2). The PCR products were digested with appropriate restriction enzymes and cloned into both pGADT7 and pGBKT7 to generate the AD (active domain) and BD (binding domain) fusion plasmids, respectively. Protein–protein interactions were carried out as described previously [35].

### Transcription and Primer Extension Assays

Run-off transcription *in vitro* assays were performed as described previously by Huang *et al* with minor modifications [21]. The template used for transcription was amplified with primers P4/P15, the mutated templates were derived from it by PCR with primers listed in Table 2, and the mutated templates were equimolar concentration in the *in vitro* transcription system. Typical reaction mixtures (25 µl) contained 1 µg template DNA, 2.5 pmol of *E.coli* core RNAP (Epicentre, USA), 50 to 60 pmol of ECF σ factor Tt-RpoE1 in transcription buffer (40 mM Tris-HCl [pH 7.5], 10 mM MgCl2, 150 mM KCl, 10 mM DTT, 0.01% TritonX−100) with 0.8 mM ATP, GTP, CTP and 5 µCi [α-^32^P]-UTP. DNA and RNAP were preincubated at 4°C for 30 min and 37°C for 8 min to allow promoter binding. Nucleotide triphosphates (NTPs) were then added, and transcription proceeded for another 8 min. The RNA transcripts were extracted with phenol-chloroform and precipitated with ethanol. The pellet was resuspended in 10 µl of urea stop solution, heated to 95°C for 3 min, and separated by 7 M urea–6% polyacrylamide gel electrophoresis and autoradiography. For assay to detect the effect of Tt-TolB on the *in vitro* transcription of Tt-RpoE1, Tt-TolB was added into the reaction system at the same time with Tt-RpoE1 or after Tt-RpoE1 and RNAP incubated for 38 min with the same concentration of Tt-RpoE1 (5 µM).

For primer extension assays, the RNA samples were obtained from the transcription reaction with or without ECF σ factor Tt-RpoE1 as described above except that [α-^32^P]-UTP was substituted with UTP. The primer P16 (Table 2) was labeled at the 5′-end with [γ-^32^P]-ATP, and was used for both DNA sequencing and primer extension as described previously [36].

### Expression and Purification of Recombinant Proteins

To overproduce the His-tagged proteins Tt-RpoE1, Tt-TolB and Tt-TolB-C, *E. coli* BL21DE3 (lysS) harboring plasmid p28Tt-RpoE1, p28Tt-TolB and p28Tt-TolB-C were cultivated in LB to an optimal density at 600 nm of 0.6 at 37°C and induced with 0.3 mM isopropyl β-D-1-thiogalactopyranoside (IPTG) at 24°C overnight. Then the proteins were extracted and purified as described previously [35]. All the purified proteins were analyzed by SDS-PAGE and the protein concentrations were determined by using the BCA™ protein concentration assay kit (PIERCE).

### Electrophoretic Mobility Shift Assay (EMSA)

Double-stranded or fork-junction probes were obtained by annealing reaction containing equimolar concentration of two oligonucleotides (Table S1) in 50 mM Tris-HCl (pH8.0), 10 mM MgCl_2_, 50 mM NaCl and 1 mM EDTA, with the top strands labeled at the 5′-end with [γ-^32^P] ATP. The 20 µl-standard binding reaction contained: 50 mM Tris-HCl (pH 8.0), 10 mM MgAc_2_, 30 mM NaCl, 1 mM DTT, 5% glycerol, 30 µg/ml BSA, 0.5 mM EDTA, 1 µg poly (dI-dC), 20 fmol labeled DNA probe and the indicated amounts of appropriate proteins. After incubation at 25°C for 20 min, samples were immediately loaded on native 5% polyacrylamide gel (mono/bis, 80∶1) in 0.5× TBE buffer and electrophoresis at 150 V for 2 h. Gels were dried and exposed to Biomax radiographic film (Kodak) for autoradiography.

## Supporting Information

Table S1 Nucleotide sequences for electrophoretic mobility shift assay.
(DOC)
