# Supplementary material for: Identification and Characterization of the Cognate Anti-Sigma Factor and Specific Promoter Elements of a T. tengcongensis ECF Sigma Factor
Source: PLoS One. 2012 Jul 16;7(7):e40885. doi: 10.1371/journal.pone.0040885 (PMC3397946; doi:10.1371/journal.pone.0040885)
Supplement: Table S1 — Nucleotide sequences for electrophoretic mobility shift assay. (DOC) [file pone.0040885.s001.doc]

**Supplementary Tables**

**Table S1** Nucleotide sequences for electrophoretic mobility shift assay

| Names | Sequences (5' to 3') | Purposes |
| --- | --- | --- |
| T+1 | GGTATTGTTACCTTTTGTGTAGATTTTCGTCTATAAAGGTG |  |
| B+1 | cacctttatagacgaaaatctacacaaaaggtaacaatacc | T+1/B+1 probe |
| B-6 | tatagacgaaaatctacacaaaaggtaacaatacc | T+1/B-6 probe |
| B-10 | gacgaaaatctacacaaaaggtaacaatacc | T+1/B-10 probe |
| B-12 | cgaaaatctacacaaaaggtaacaatacc | T+1/B-12 probe |
| B-13 | gaaaatctacacaaaaggtaacaatacc | T+1/B-13 probe |
| D-5  D-5-Bot.* | tgttaccttttgtgtagattttcgtctataaaggtg  gacgaaaatctacacaaaaggtaaca | D-5/-10 probe, truncation from -40 to -36 |
| D-9  D-9-Bot. | accttttgtgtagattttcgtctataaaggtg  gacgaaaatctacacaaaaggt | D-9/-10 probe, truncation from -40 to -32 |
| D-12  D-12-Bot. | ttttgtgtagattttcgtctataaaggt  gacgaaaatctacacaaaa | D-12/-10 probe, truncation from -40 to -30 |
| D-5-1T  D-5-1T-Bot. | GGttaccttttgtgtagattttCGtctataaaggtg  gacgaaaatctacacaaaaggtaacc | substitution at -35T |
| D-5-2G  D-5-2G-Bot. | tTttaccttttgtgtagattttCGtctataaaggtg  gacgaaaatctacacaaaaggtaaAa | substitution at -34G |
| D-5-3t  D-5-3t-Bot. | tgGtaccttttgtgtagattttcgtctataaaggtg  gacgaaaatctacacaaaaggtaCcA | substitution at -33T |
| D-5-4t  D-5-4t-Bot. | tgtGaccttttgtgtagattttcgtctataaaggtg  gacgaaaatctacacaaaaggtCaca | substitution at -32T |
| D-5-5a  D-5-5a-Bot. | tgttGccttttgtgtagattttcgtctataaaggtg  gacgaaaatctacacaaaaggCaaca | substitution at -31A |
| D-5-6c  D-5-6c-Bot. | tgttaTcttttgtgtagattttcgtctataaaggtg  gacgaaaatctacacaaaagAtaaca | substitution at -30C |
| D-5-7c  D-5-7c-Bot. | tgttactttttgtgtagattttcgtctataaaggtg  gacgaaaatctacacaaaaagtaaca | substitution at -29C |
| D-5- M1  D-5-M1-Bot. | tgGGaccttttgtgtagattttCGtctataaaggtg  gacgaaaatctacacaaaaggtCCca | substitution at -(33-32) |
| D-5-M2  D-5-M2-Bot. | gtttaccttttgtgtagattttCGtctataaaggtg  gacgaaaatctacacaaaaggtaaac | substitution at -(35-34) |
| D-5- M3  D-5-M3-Bot. | gtGGaccttttgtgtagattttCGtctataaaggtg  gacgaaaatctacacaaaaggtCCac | substitution at -(35-32) |
| D-5-M4  D-5-M4-Bot. | tgtGgccttttgtgtagattttcgtctataaaggtg  gacgaaaatctacacaaaaggcCaca | substitution at -(32-31) |
| D-5-M5  D-5-M5-Bot. | tgtGgTTttttgtgtagattttcgtctataaaggtg  gacgaaaatctacacaaaaAAcCaca | substitution at -(32-29) |
| D-5-B1C  D-5B1C-Bot. | tgttaccttttgtgtagattttTgtctataaaggtg  CAaaaatctacacaaaaggtaaca | substitution at -13C |
| D-5-B2G  D-5B2G-Bot. | tgttaccttttgtgtagattttcTtctataaaggtg  AGaaaatctacacaaaaggtaaca | substitution at -12G |
| D-5-B3T | tgttaccttttgtgtagattttcgccTAtaaaggtg | D-5-B3T/-12 for substitution at -11T |
| D-5-b4c | tgttaccttttgtgtagattttcgtttataaaggtg | D-5-b4c -12 for substitution at -10C |
| D-5-b5t | tgttaccttttgtgtagattttcgtcgataaaggtg | D-5-b5t /-12 for substitution at -9T |
| D-5-b6a | tgttaccttttgtgtagattttcgtctgtaaaggtg | D-5-b6a/-12 for substitution at -8A |
| D-5-b7t | tgttaccttttgtgtagattttcgtctagaaaggtg | D-5-b7t/-12 for substitution at -7T |
| D-5-b8a | tgttaccttttgtgtagattttcgtctatgaaggtg | D-5-b8a/-12 for substitution at -6A |
| D-5-BM1  D-5BM1-Bot. | tgttaccttttgtgtagattttTTtctata aaggtg  AAaaaatctacacaaaaggtaaca | substitution at –(13-12) |
| D-5-BM2  D-5BM2-Bot. | tgttaccttttgtgtagattttTTtTtataaaggtg  AAaaaatctacacaaaaggtaaca | substitution at –(13,12,10) |
| D-5-BM3 | tgttaccttttgtgtagattttcgTcCCTAaaggtg | substitution at –(9-8) |
| D-5-BM4 | tgttaccttttgtgtagattttcgTcCCCCaaggtg | substitution at –(9-6) |

___________________________________________________________________________________________________________________________

* Bot., abbreviation of Bottom strand.
